# Population genomics of two congeneric Palaearctic shorebirds reveals differential impacts of Quaternary climate oscillations across habitats types

**DOI:** 10.1038/s41598-019-54715-9

**Published:** 2019-12-03

**Authors:** Hui Zhen Tan, Elize Ying Xin Ng, Qian Tang, Gary A. Allport, Justin J. F. J. Jansen, Pavel S. Tomkovich, Frank E. Rheindt

**Affiliations:** 1 Department of Biological Sciences, National University of Singapore, 16 Science Drive 4, Singapore, 117558 Singapore; 2 BirdLife International, The David Attenborough Building, Pembroke Street, Cambridge, CB2 3QZ UK; 3 Naturalis Biodiversity Center, Leiden, P.O. Box 9517, 2300 RA Leiden, The Netherlands; 4 Zoological Museum, Lomonosov Moscow State University, Bolshaya Nikitskaya Str. 2, Moscow, 125009 Russia

**Keywords:** Molecular ecology, Ecological genetics, Biogeography

## Abstract

Intracontinental biotic divisions across the vast Palaearctic region are not well-characterized. Past research has revealed patterns ranging from a lack of population structure to deep divergences along varied lines of separation. Here we compared biogeographic patterns of two Palaearctic shorebirds with different habitat preferences, Whimbrel (*Numenius phaeopus*) and Eurasian curlew (*N. arquata*). Using genome-wide markers from populations across the Palaearctic, we applied a multitude of population genomic and phylogenomic approaches to elucidate population structure. Most importantly, we tested for isolation by distance and visualized barriers and corridors to gene flow. We found shallow Palaearctic population structure in subpolar bog and tundra-breeding whimbrels, consistent with other species breeding at a similarly high latitude, indicating connectivity across the tundra belt, both presently and during southward shifts in periods of global cooling. In contrast, the temperate grassland-breeding Eurasian curlew emerged in three distinct clades corresponding to glacial refugia. Barriers to gene flow coincided with areas of topographic relief in the central Palaearctic for whimbrels and further east for Eurasian curlews. Our findings highlight the interplay of historic and ecological factors in influencing present-day population structure of Palaearctic biota.

## Introduction

Extreme climatic events in the Earth’s recent past have played an important role in shaping population genetic structure of animals^1,2^. It is surmised that the impact of Quaternary climate oscillations on biogeography differs according to a suite of variables, such as latitude and topography^2^. During cyclical periods of global cooling, the formation of barriers has facilitated intercontinental biotic differentiation as revealed by traditional DNA markers^3–7^. While intra-continental population structure is equally impacted by Quaternary climate oscillations, it has not been studied as intensely, with most research focusing on North America and Europe^8–11^. The Nearctic and Palaearctic, both large northern hemispheric landmasses, are characterized by a zoogeographical division into an eastern and a western sub-region. The boundary between North America’s sub-regions runs along a suture zone through the center of the Great Plains and has been well described^12–20^. In the Palaearctic, a vast zoogeographic region stretching across the world’s most sizeable landmass, Eurasia^21,22^, the large-scale division into western and eastern sub-regions has long been established based on the distinct faunal elements present on either side^23,24^. However, resolution of the boundary of these two sub-regions across biota has so far eluded biogeographers.

Novel population genomic approaches relying on massive panels of genome-wide loci afford an opportunity to identify intra-Palaearctic barriers and contrast them among different species. The last two decades have seen an increase in molecular studies on population genetic divisions of wide-ranging species complexes, revealing a variety of phylogeographic patterns traversing the Palaearctic^25–28^. Population genetic inquiries on many vertebrates^29–31^, especially songbirds^3,25,28,32–35^, and invertebrates^36,37^ have revealed a primary divide between Far-Eastern populations (east of Lake Baikal to Japan) and the much more expansive western and central Palaearctic populations. An alternative dividing line within wide-ranging Palaearctic species has been proposed further west in the Central Palaearctic^27,28,38,39^. In other studies, Palaearctic populations have also been characterized by divisions into multiple clusters corresponding to glacial refugia during the Last Glacial Maximum (LGM)^40,41^. Conversely, some wide-ranging species lack or show only shallow population structure despite the vast geographical area involved^9,25,28,31,42–44^. Explanations for these phylogeographic patterns often invoke geographical features and environmental conditions both presently and historically^44^, which interact with life-history traits such as dispersal capability^9^, natal homing^45^, prey availability^42^, non-breeding habitat preference^46^, mating system^47–49^ as well as the evolutionary history unique to each species^28^. This complicated interplay of factors accounts for substantial variability in the boundary between eastern and western Palaearctic population units across species.

**Figure 1:**
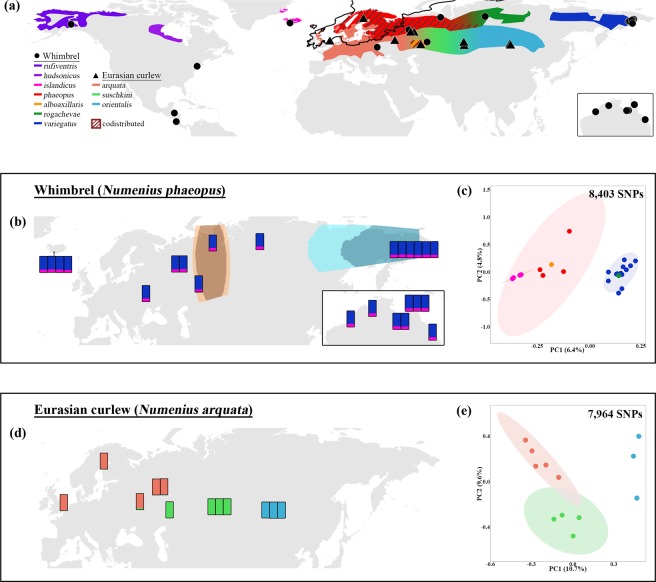
(**a**) Distribution of breeding areas and sampling localities of whimbrel (*Numenius phaeopus*) and Eurasian curlew (*N. arquata*). Each circle or triangle symbol represents a sampled individual. The black lines across Europe represent the maximum extent of the European ice sheet at the last glacial maximum^70^. (**b**) Coloured bars represent Structure results at K = 2 for Palaearctic whimbrels. Each bar represents the results for an individual at its approximate sampling locality. Orange and blue polygons represent barriers and corridors to gene flow, respectively, as identified by EEMS. Dark and light shades represent posterior probabilities of >0.95 and >0.90, respectively. The inset shows the results for whimbrels sampled from Australia. (**c**) Principal component (PC) analysis of Palaearctic whimbrels, with percentage of variation of the two most important PCs. Ellipses represent 95% confidence intervals. Due to low sample size, no ellipses were calculated for *N. p. alboaxillaris*, and *rogachevae*. Colours correspond to the breeding populations in (**a**). (**d**) Coloured bars represent Structure results at K = 3 for Eurasian curlews. Each bar represents the results for an individual at its approximate sampling locality. No significant barriers or corridors were identified by EEMS. (**e**) PC analysis of Eurasian curlews, with percentage of variation of the two most important PCs. Ellipses represent 95% confidence intervals. Due to low sample size, no ellipses were calculated for *N. a. orientalis*. Colours correspond to the breeding populations in (**a**).

There continues to be a dearth of comparative research on Palaearctic biota across different habitat types to help shed light on the historic mechanisms that have shaped their evolutionary histories^2^. In this study, we used thousands of genome-wide markers to elucidate fine-scale population structure across the Palaearctic in two migratory shorebirds – the whimbrel (*Numenius phaeopus* Linnaeus, 1758) and Eurasian curlew (*Numenius arquata* Linnaeus, 1758). The Eurasian curlew breeds in temperate grassy mires and wet meadows, with a relatively short-distance migratory behavior. Contrarily, the whimbrel generally breeds in extensive bogs in woodlands and tundra of subpolar latitudes, predominantly wintering at (sub-)tropical latitudes (Fig. 1a). An exception is the steppe whimbrel, *N. p. alboaxillaris*, which breeds in wet grasslands in steppe valleys sympatrically with Eurasian curlew. Both species are polytypic, ranging from the western to eastern Palaearctic and exhibiting intraspecific plumage variation. In both species, populations breeding in the South Urals (steppe whimbrel and steppe curlew, *N. a. suschkini*) are phenotypically distinct as the palest taxa^50–53^. By comparing the phylogeography between two congeneric shorebird species with a similar biology but important differences in breeding habitat, we aim to shed light on habitat-specific effects of Quaternary climate oscillations on biotic differentiation across the Palaearctic.

## Results

### Sequencing and single nucleotide polymorphism (SNP) harvest

A total of 54 double digest restriction-site associated DNA sequencing (ddRADseq) libraries spanning 38 whimbrels, 15 Eurasian curlews and one common redshank were successfully prepared (see Supplementary Table S1), amounting to a total of 162,836,371 paired-end 150 bp Illumina sequence reads. Thirteen samples (10 whimbrels and 3 Eurasian curlews) were excluded from downstream analysis due to low coverage or more than 25% missing data. We obtained between 400,000–17,000,000 reads per individual and harvested between 6,500–8,500 SNPs across population genomic datasets (see Supplementary Table S2). We obtained 438,477 bp of data for phylogenomic analysis of all whimbrels.

### Whimbrel phylogeography

**Figure 2:**
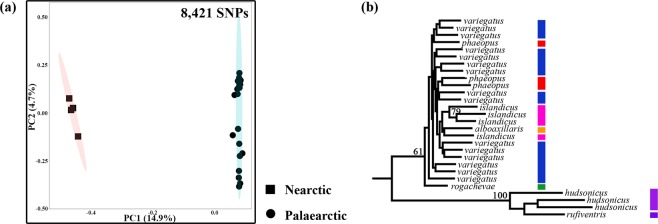
(**a**) Principal component (PC) analysis of all whimbrels, with percentage of variation for the two most important PCs, including Nearctic (*N. p. hudsonicus* and *rufiventris*) and Palaearctic (*N. p. phaeopus*, *islandicus*, *alboaxillaris*, *rogachevae* and *variegatus*) populations. Ellipses represent 95% confidence intervals. (**b**) Maximum likelihood tree of all whimbrels using 438,477 bp of sequence data. Only bootstrap support >50 is displayed. Colours of the bars at the terminal ends of branches correspond to the breeding populations in Fig. 1a.

Palaearctic whimbrels formed a well separated cluster from Nearctic whimbrels (Fig. 2a). Phylogenetic analysis also showed that Nearctic whimbrels formed a deep monophyletic clade with high bootstrap support (100%) (Fig. 2b). In summary, analysis across all whimbrel individuals (n = 28) revealed two distinct monophyletic groups comprising Nearctic versus Palaearctic populations.

Within Palaearctic whimbrels, no noticeable geographic differentiation was apparent in Structure analysis at any values of K ranging from K = 1 to 8 (Fig. 1b; see Supplementary Fig. S1). This uniformity suggests that Palaearctic whimbrel populations are connected by substantial gene flow, belying the vast geographic distances among populations. When conducting Structure analysis at increasing K, there is indication of clinality between eastern (*variegatus*, *rogachevae*) and western (*phaeopus*, *islandicus*, *alboaxillaris*) populations (see non-blue parts of Structure plot in Supplementary Fig. S1), suggesting possible isolation by distance (see Supplementary Fig. S1) as confirmed by a significant Mantel’s paired test (r = 0.414, p-value ≤ 0.001).

Estimated Effective Migration Surfaces (EEMS) analysis identified a barrier of low effective migration overlapping with and slightly east of the continental divide between Europe and Asia, indicating an area where the decay of genetic similarity with increasing geographic distance is significantly higher than expected under exact isolation-by-distance (Fig. 1b). The barrier divides eastern and western Palaearctic populations, which was also reflected as the main genomic division in principal component analysis (PCA) along principal component 1 (PC1) (Fig. 1c). However, this divergence only accounts for 6.4% of total genomic variation in PCA, alluding to generally low genomic differentiation among all Palaearctic whimbrels.

The most recently described whimbrel subspecies *rogachevae* from eastern Evenkia, central Siberia, was embedded with the Far-Eastern *variegatus* and both populations are potentially connected through a corridor of high effective migration (Fig. 1b,c). However, we only sampled one individual of *rogachevae* and additional sampling is required to validate, and furthermore resolve, the location of the effective migration corridor. Our sole sample of the pale steppe whimbrel was embedded within the western clade (Fig. 1c).

### Eurasian curlew phylogeography

In contrast to the whimbrels, three distinct Eurasian curlew populations emerged, each corresponding to established subspecies^51^ (Fig. 1d; K = 3, ideal number of clusters^54^). At other values of K, the three Eurasian curlew populations remained discrete (see Supplementary Fig. S2). The main division was between eastern *orientalis* versus the more westerly *suschkini* and *arquata*, accounting for 10.7% of variation along PC1 (Fig. 1e).

A Mantel’s paired test failed to reveal a significant correlation (r = 0.173, p-value = 0.137) between genetic and geographic distance in Eurasian curlews. Further investigation into isolation by distance using EEMS analysis, accordingly, found no evidence for significant corridors or barriers within the Eurasian curlew’s range (see Supplementary Fig. S3). The absence of isolation by distance is typical for analyses that include deeply differentiated populations.

As in whimbrels, the affinity of the steppe curlew from the South Urals was closer to European populations (Fig. 1e).

## Discussion

Our genome-wide data corroborate previous mtDNA-based studies^4,6,26,55^ in that Nearctic whimbrel populations are deeply differentiated from Palaearctic populations. Populations on the two continents also have a fixed difference in rump colouration^52,56^. This lack of substantial gene flow is despite opportunities when land bridges have connected Asia and North America^57,58^. We found no evidence for genomic admixture between Nearctic and Palaearctic whimbrels via *variegatus*, the Far-Eastern Palaearctic subspecies (Fig. 1a) conjectured to be an intermediate form between continents^59^. Genome-wide, mitochondrial and plumage evidence points to a deep rift between Nearctic and Palaearctic populations, advocating the elevation of North American breeding populations to species level as the “Hudsonian whimbrel” *N. hudsonicus*^6,52^.

Within Palaearctic whimbrels, a general lack of differentiation (Fig. 1b; see Supplementary Fig. S1) suggests extensive continent-wide gene flow as in other shorebirds^28,48,49,60–62^ and terrestrial vertebrates^9,25,31,42,44,63^. Palaeoclimatic habitat reconstructions of the LGM attest to a broad and nearly unbroken belt of suitable habitat stretching along the entire southern margin of the North European Ice Sheet^64,65^, which would have facilitated genetic exchange. Species breeding at high latitudes face displacement by advancing ice sheets during climatic oscillations^66,67^ and the relatively dynamic environment impedes isolation and differentiation of populations^68^. Conversely, the largely unglaciated expanses of the Far-Eastern Palaearctic have been a refuge from which species later re-expanded^9,36,69–71^ resulting in the divergence of East Asian populations^37,66^.

In contrast, the Eurasian curlew emerged as three distinct populations (Fig. 1d; see Supplementary Fig. S2) corresponding to three recognized subspecies. Such deeper population division is consistent with a large body of work on temperate biota showing genomic signatures of population re-expansions from separate glacial refugia^32,34,40,72^. Identifying the locations of these refugia would require more extensive sampling from across the curlew’s range. In addition, the migration pattern of Eurasian curlews also favours a reduction of population mixing and facilitates their accumulation of genetic differences. Eurasian curlews are shorter-distance migrants and are known to exhibit high fidelity to breeding sites^73^. On the other hand, whimbrels move over larger distances during migration (e.g., from East Siberia to Australia) and may disperse more widely among different breeding populations^74^. Previous shorebird studies have found mating system and migratory strategy to have an effect on population differentiation^46–48^. These are, however, not pertinent to differences in genetic structure between whimbrels and Eurasian curlews as both species are monogamous and overlap in non-breeding habitats^73,74^.

In both species, we found no evidence for a deep genomic separation of the palest taxa breeding in the South Urals, steppe whimbrel *alboaxillaris* and steppe curlew *suschkini*, from their respective conspecifics. However, only a single steppe whimbrel sample with moderate sequence coverage was available and deeper sampling will be needed for more authoritative statements on its genomic distinctness. The steppes south of the Urals are arid and experience warm summers^75^. The pale plumages of these subspecies are consistent with Gloger’s Rule^76^, which predicts that more arid environments harbour less heavily pigmented populations. The discordance between morphological and genetic differentiation may indicate a rapid evolution of ecomorphological adaptations controlled by few genes^77^ or phenotypic plasticity in response to environmental conditions^78–83^. The steppe whimbrel is now known to be an exceedingly rare taxon^50,53,84^. Even if future research upholds a lack of deep genomic differentiation in steppe whimbrels, their distinct plumage still warrants conservation efforts to preserve unique ecomorphological adaptations^50,85^.

Investigations into patterns of gene flow using EEMS analysis identified a barrier between the subspecies *rogachevae* from central Siberia and *phaeopus* breeding in sub-Arctic Europe (Fig. 1b). These results refute previous plumage-based predictions that *variegatus* is the most deeply differentiated Palaearctic whimbrel taxon on account of its dense rump barring^6,59^, but point to the importance of differences in axillary coloration uniting *rogachevae* and *variegatus* into an eastern cluster distinct from western *phaeopus*^86^.

The location of the EEMS barrier approximately overlaps with the Urals, a mountain range separating Europe from Asia, whose topographic relief may have rendered its slopes unsuitable for *Numenius*. The Urals may act as a suture zone in which phylogeographic breaks cluster^20,39,87,88^. Alternatively, the Yenisey area of Siberia has been proposed as an important zoogeographical boundary^89^, although – in the context of whimbrels – it lies to the east of the population divide identified by EEMS. It involves a vast transitional area with natural zonation amongst different habitats and was initially identified as the area where populations with typical *phaeopus* plumage transition into a *rogachevae* plumage type^86^.

As opposed to whimbrels, Eurasian curlews are temperate grassland and marsh breeders. The primary division in our Eurasian curlew dataset is likely deeper than that in whimbrels, and located further east, running between the subspecies *suschkini* and *orientalis* (Fig. 1d,e; see Supplementary Figs. S2 and S3). This phylogeographic break falls within the Altai and Sayan Mountains separating open steppe habitats of Central Asia from the grasslands and river marshes of southern-central Siberia, northern Mongolia, Buryatia and the Amur region. Therefore, this barrier again coincides with areas of significant topographic relief that are unsuitable for curlew breeding, even during periods of global cooling. Ice-dammed lakes flooded parts of Russia during the LGM and may have posed a barrier to dispersal, especially those formed by the Ob River^90^. In summary, our analyses attest to the differential impact that Quaternary climate oscillations have had on biota with different habitat preferences.

## Methods

### Sampling regime

Our sampling regime aimed at a complete representation of all named taxa of the whimbrel and Eurasian curlew. A total of 53 tissue (muscle or liver) and blood samples were loaned (whimbrel: 38, Eurasian curlew: 15; see Supplementary Table S1). We assigned specimens lacking in subspecies identification based on sampling locality and known breeding distributions^86,91,92^. Distribution of the eastern *N. a. orientalis* may extend further west to intergrade with the western *arquata*^51^ (Fig. 1a) but ranges are not well resolved due to a lack of studies. Whimbrels from wintering localities in the Nearctic were not assigned while those in the Palaearctic were assigned to *N. phaeopus variegatus* based on their Australian provenance; there were no Eurasian curlew individuals from wintering localities. A common redshank *Tringa totanus* sample was included as an outgroup for phylogenetic rooting.

### Library preparation, sequencing and raw data processing

DNA extractions were performed with the DNEasy Blood & Tissue Kit (Qiagen, Hilden, Germany) with an additional incubation step with heat-treated RNase. We prepared a reduced representation library using a modified ddRADSeq protocol^93,94^. Electrophoretic size selection for DNA fragments of 350 bp (±31 bp) was performed with Pippin Prep (Sage Science, Beverly, US). Pools were combined at equimolar volumes. The final library was spiked with 30% phiX and 150 bp paired-end reads were sequenced on a HiSeq. 4000 Illumina platform (Genome Institute of Singapore).

We checked the accuracy of sequencing of each base via phred scores with FastQC 0.11.5 (https://www.bioinformatics.babraham.ac.uk/projects/fastqc/). As all bases had a mean phred score >30 (≥99.9% base call accuracy), no truncation was necessary. Raw sequences were demultiplexed and cleaned using process_radtags in Stacks 1.44^95^. We mostly discarded reads with uncalled bases (*–c*) but rescued reads if their barcodes contained two or fewer mismatches from the barcodes provided (*–r*). Only samples with more than 400,000 reads were retained for downstream analysis. We then aligned sequences to the Ruff *Calidris pugnax* genome^96,97^ using BWA-MEM 0.7.1^98,99^ to identify homologous regions. Alignment quality was checked with samtools flagstat and sorted according to coordinate order with samtools sort in samtools 1.3.1^100^.

### SNP calling

We created four SNP datasets for population genomic analysis: (1) all whimbrels, (2) all Palaearctic whimbrels, (3) Palaearctic whimbrels in breeding areas only, and (4) all Eurasian curlews. Loci were identified from sequences aligned to the reference genome using ref_map.pl in Stacks, followed by filtering using populations. We retained loci present in 90% of individuals (*–r*) and with a stack depth (minimum number of reads, *–m*) of 10 and 5 for Eurasian curlews and whimbrels, respectively (see Supplementary Table S2). To avoid obtaining linked SNPs, only the first SNP in each fragment was called and then filtered to remove linkage disequilibrium (r^2^ threshold of 0.9) using PLINK 1.9^101^. Using PLINK, we also quantified missing data per individual. Relatedness analysis was conducted to estimate identity by descent using maximum-likelihood (ML) estimation in ‘SNPRelate’ as implemented in R 3.5.1^102,103^ (see Supplementary Table S3). Individuals with >30% missing data were removed and SNPs were re-called from ref_map.pl. We checked SNP loci for neutrality in BayeScan 2.1^104^ using default settings. At a 5% false discovery rate, all SNPs showed no apparent signatures of selection and were retained.

We aimed to resolve the genomic affinity of the palest populations breeding in the South Urals. However, one of the taxa in question, the steppe whimbrel, was only represented by one individual with moderate sequence coverage. Hence, we partitioned this individual from other whimbrels (*–p* 2) during SNP calling in populations. This practice mitigated low coverage and missing data in this sample and ensured that all loci identified would be informative for this taxon of interest. No population partitioning was implemented for Eurasian curlews during SNP calling.

### Population genomic structure analysis

To investigate population structure, we conducted PCA on three datasets: all whimbrels, only Palaearctic whimbrels, and Eurasian curlews, using ‘SNPRelate’. Population structure within Palaearctic populations of both species was further investigated in Structure 2.3^105^. An admixture model was applied and five iterations of each K from K = 1 to K = 10 at most were run with 50,000 burn-in cycles and 250,000 Markov Chain Monte Carlo (MCMC) simulations. Consensus structure results for each K value were obtained using CLUMPP 1.1.2^106^ and an optimal number of clusters was inferred where required^54^.

For analyses involving geographic information, only individuals from breeding localities in the Palaearctic were included. We tested for isolation by distance using a Mantel’s test with 999 replicates in ‘poppr’. We also implemented EEMS^107^ by performing three independent chains of 8 million MCMC iterations with a 1 million iteration burn-in using 200, 400 and 600 demes. Results were checked for consistency across the different regimes implemented and for convergence of MCMC runs. Finally, results across runs were combined and visualized using ‘rEEMSplots’^107^.

### Phylogenomic analysis

We employed PyRAD 3.0.64^108^ to identify sequence data for phylogenomic analysis of all whimbrels^109^. The ddRADseq loci were assembled *de novo* using only the first read in each pair of paired-end sequences (*ddrad*) and an overall clustering threshold of 0.88 was applied. Loci had to be present in 90% of individuals (*MinCov* 26) with a minimum coverage of five per cluster (*MinDepth* 5) and a maximum of four undetermined (“N”) sites. A maximum of three shared polymorphic sites per locus was allowed (*maxSH* 3) to avoid inclusion of paralogs with fixed differences.

A ML tree was constructed for whimbrels in RAxML version 8.2.9^110^ using concatenated sequence reads identified by PyRAD. We applied two general time reversible models, with an optimisation of the substitution rate under a gamma distribution and a site-specific optimization of the substitution rate. A total of 1000 alternative trees were constructed in a rapid bootstrap analysis. The model with the lowest Akaike Information Criterion was selected and the best-scoring ML tree was visualised using Mesquite 3.2^111^.

## Supplementary information

Supplementary information

## Data Availability

The data generated in this study will be available in the Sequence Read Archive repository (BioProject Number: PRJNA562783).
