# Supplementary material for: Population genomics of two congeneric Palaearctic shorebirds reveals differential impacts of Quaternary climate oscillations across habitats types
Source: Sci Rep. 2019 Dec 3;9:18172. doi: 10.1038/s41598-019-54715-9 (PMC6890745; doi:10.1038/s41598-019-54715-9)
Supplement: Supplementary file 1 — Supplementary information [file 41598_2019_54715_MOESM1_ESM.docx]

**­­Supplementary information**

#

# **Population genomics of two congeneric Palaearctic shorebirds reveals differential impacts of Quaternary climate oscillations across habitats types**

Tan, Hui Zhen, Ng, Elize Ying Xin, Tang, Qian, Allport, Gary A., Jansen, Justin J. F. J., Tomkovich, Pavel S., Rheindt, Frank E.

**Supplementary Table S1.** Details of samples including locality and year collected, museum loaned from (AMNH – American Museum of Natural History; ANWC – Australian National Wildlife Collection; BMUW – Burke Museum; LKCNHM – Lee Kong Chian Natural History Museum; UOI – University of Iceland; ZMMU – Zoological Museum of Moscow State University) and the final DNA sequence read numbers and coverage. Samples highlighted in grey were not included in downstream bioinformatic analyses due to relatively low number of sequence reads or high missing data. Sequenced reads will be available in the Sequence Read Archive repository (BioProject Number: PRJNA562783).

| Taxon | | | | Sample | Locality | Country | Year | Museum | Sequence Reads | Coverage |
| --- | --- | --- | --- | --- | --- | --- | --- | --- | --- | --- |
| Whimbrel (*Numenius phaeopus*) | Palaearctic | Western | *phaeopus* | WP1 | Lala River, Kirov | Russia | 2003 | ZMMU | 42,398 | - |
|  |  |  |  | WP2 | Border with Pskov, Vitebsk | Belarus | 2012 | ZMMU | 2,554,670 | 47.2 |
|  |  |  |  | WP3 | Nyarovej Khadyta River, Tyumen | Russia | 2007 | ZMMU | 298,523 | - |
|  |  |  |  | WP5 | Curonian Spit, Kaliningrad | Russia | 2005 | ZMMU | 115,791 | - |
|  |  |  |  | WP6 | Ul’skoe Mire, Kirov | Russia | 2003 | ZMMU | 684,453 | 12.3 |
|  |  |  |  | WP7 | Ul’skoe Mire, Kirov | Russia | 2003 | ZMMU | 2,096,543 | 25.2 |
|  |  |  |  | WP8 | Labytnangi,  Yamalo-Nenetskiy Avtonomnyy Okrug | Russia | 1997 | BMUW | 6,847,811 | 28.9 |
|  |  |  | *islandicus* | WD1 | Southern Region | Iceland | 2016 | UOI | 6,393,303 | 95.8 |
|  |  |  |  | WD2 | Southern Region | Iceland | 2016 | UOI | 271,980 | - |
|  |  |  |  | WD3 | Southern Region | Iceland | 2016 | UOI | 5,316,138 | 63.3 |
|  |  |  |  | WD4 | Southern Region | Iceland | 2016 | UOI | 6,827,511 | 105.9 |
|  |  |  |  | WD5 | Southern Region | Iceland | 2016 | UOI | 3,447,133 | 58.6 |
|  |  |  | *alboaxillaris* | WA3 | Blagoveshchenskaya,  Krasnodarskiy Kray | Russia | 1998 | BMUW | 2,050,243 | - |
|  |  |  |  | WA6 | Maly Kizel Lake, Bashkortastan | Russia | 2007 | ZMMU | 471,825 | 11.8 |
|  |  | Eastern | *rogachevae* | WC4 | Severnoe Lake, Krasnoyarsk | Russia | 2003 | ZMMU | 5,422,825 | 53.7 |
|  |  |  | *variegatus* | WV1 | Sea of Karumba,  Queensland | Australia | 2001 | ANWC | 3,115,203 | 48.7 |
|  |  |  |  | WV2 | Albert River, Queensland | Australia | 2001 | ANWC | 386,036 | 62.6 |
|  |  |  |  | WV3 | Victoria River Mouth, Northern Territory | Australia | 2003 | ANWC | 4,129,725 | 57.0 |
|  |  |  |  | WV4 | Shoalwater Bay Army Training Reserve, Queensland | Australia | 1992 | ANWC | 3,620,522 | 57.1 |
|  |  |  |  | WV5 | Thangoo Station, Western Australia | Australia | 2004 | ANWC | 4,114,190 | 53.6 |
|  |  |  |  | WV6 | Kalpowar Station, Queensland | Australia | 2006 | ANWC | 3,456,003 | 53.9 |
|  |  |  |  | WV7 | Kalpowar Station, Queensland | Australia | 2006 | ANWC | 7,193,641 | 107.0 |
|  |  |  |  | WV8 | Kalpowar Station, Queensland | Australia | 2006 | ANWC | 2,522,302 | 45.1 |
|  |  |  |  | WV9 | Sungei Buloh Wetland Reserve | Singapore | 1992 | AMNH | 347,346 | - |
|  |  |  |  | WV10 | Kanchalan River, Chukotsky | Russia | 2006 | ZMMU | 992,833 | 19.5 |
|  |  |  |  | WV11 | Meinypil’gyno, Chukotsky | Russia | 2011 | ZMMU | 4,426,125 | 50.0 |
|  |  |  |  | WV12 | Krasneno, Chkotsky | Russia | 2006 | ZMMU | 1,622,103 | 30.2 |
|  |  |  |  | WV13 | Telegraphny Cape, Chukotsky | Russia | 2006 | ZMMU | 604,123 | 14.1 |
|  |  |  |  | WV14 | Kanchalan River, Chukotsky | Russia | 2005 | ZMMU | 4,439,558 | 47.3 |
|  |  |  |  | WV15 | Meinypil’gyno, Chukotsky | Russia | 2009 | ZMMU | 373,914 | - |
|  |  |  |  | WV16 | Meinypil’gyno, Chukotsky | Russia | 2011 | ZMMU | 2,637,332 | 46.3 |
|  | Nearctic | | *hudsonicus* | WH1 | JFK Airport, New York | USA | 2004 | AMNH | 3,180,271 | 42.4 |
|  |  |  |  | WH2 | La Flor, Departamento de Rivas | Nicaragua | 1996 | BMUW | 7,715,966 | 81.5 |
|  |  |  |  | WH3 | La Ceiba, Departmento de Atlantida | Honduras | 2001 | BMUW | 3,778,383 | 58.3 |
|  |  |  | *rufiventris* | WF1 | Middleton Island, Alaska | USA | 2014 | AMNH | 24,138 | - |
|  |  |  |  | WF2 | Paxson, Alaska | USA | 1995 | BMUW | 6,451,249 | 71.6 |
|  |  |  |  | WF3 | Westport, Washington State | USA | 1989 | BMUW | 16,199 | - |
|  |  |  |  | WF4 | Coffee Dome, Alaska | USA | 2006 | ZMMU | 378,121 | - |
| Eurasian curlew (*Numenius arquata*) | Central/ Western | | *arquata* | EA1 | Barrow-in-Furness, England | UK | 1970 | AMNH | 4,154,529 | 79.9 |
|  |  |  |  | EA2 | Angesbyn, Norrbotten | Sweden | 2005 | AMNH | 1,785,472 | 36.3 |
|  |  |  |  | EA3 | Semion, Ryazen | Russia | 2008 | ZMMU | 1,082,809 | 27.0 |
|  |  |  |  | EA4 | Beregovoh, Kirov | Russia | 2002 | ZMMU | 409,003 | - |
|  |  |  |  | EA9 | Tcheptsa river, Kirovskaya Oblast | Russia | 2002 | BMUW | 2,808,038 | 54.9 |
|  |  |  |  | EA10 | Strizhy, Kirovskaya Oblast | Russia | 1998 | BMUW | 17,624,365 | 35.3 |
|  |  |  | *suschkini* | ES2 | Mikhailovka, Altai Krai | Russia | 2007 | ZMMU | 2,355,596 | 51.2 |
|  |  |  |  | ES3 | Mikhailovka, Altai Krai | Russia | 2007 | ZMMU | 2,097,630 | 38.7 |
|  |  |  |  | ES6 | Orenburg | Russia | 2007 | ZMMU | 54,283 | - |
|  |  |  |  | ES7 | Orenburg | Russia | 2008 | ZMMU | 1,547,835 | 35.9 |
|  | Eastern | | *orientalis* | EO1 | Mursino, Avtonomnaya Respublika Buryatiya | Russia | 2002 | BMUW | 4,179,953 | 93.5 |
|  |  |  |  | EO2 | Lyapunikha Lake, Altai Krai | Russia | 2007 | ZMMU | 1,341,706 | 33.9 |
|  |  |  |  | EO3 | Lyapunikha Lake, Altai Krai | Russia | 2007 | ZMMU | 333,427 | - |
|  |  |  |  | EO4 | Ulan-Ude, Avtonomnaya Respublika Buryatiya | Russia | 1993 | BMUW | 2,922,544 | 65.9 |
|  |  |  |  | EO5 | Ulan-Ude, Avtonomnaya Respublika Buryatiya | Russia | 1993 | BMUW | 5,050,287 | 111.1 |
| Outgroup:  Common redshank (*Tringa totanus*) | | | | TT1 | Sungei Buloh Wetland Reserve | Singapore | 2011 | LKCNHM | 6,722,464 | - |

**Supplementary Table S2.** Parameters used in SNP calling and resulting SNP numbers for all datasets: all whimbrels, all Palaearctic whimbrels, Palaearctic whimbrels in breeding areas only, and all Eurasian curlews.

| **Dataset** | **Number of individuals** | **Stack depth (–m)** | **Average reads per individual** | **Mean coverage** | **Mean missing data** | **Number of SNPs** | |
| --- | --- | --- | --- | --- | --- | --- | --- |
|  |  |  |  |  |  | **Pre-filtering** | **Final** |
| All whimbrels (population genomic) | 28 | 5 | 3,730,278 | 51.7 | 2.5% | 8,806 | 8,421 |
| Palaearctic whimbrels | 24 | 5 | 3,471,746 | 49.8 | 2.8% | 8,816 | 8,403 |
| Palaearctic whimbrels (breeding areas only) | 16 | 5 | 3,424,018 | 44.4 | 2.1% | 7,123 | 6,653 |
| Eurasian curlews | 12 | 10 | 3,912,564 | 55.3 | 2.4% | 8,742 | 7,964 |

**Supplementary Table S3**. Kinship values of sample pairs calculated in ‘SNPRelate’. Only pairs with kinship values > 0 are presented.

| **ID1** | **ID2** | **Kinship** |
| --- | --- | --- |
| WD1 | WD5 | 0.051269 |
| WD4 | WD5 | 0.049788 |
| WD1 | WD3 | 0.047024 |
| WD1 | WD4 | 0.046738 |
| WD3 | WD5 | 0.045922 |
| WD3 | WD4 | 0.044200 |
| WD1 | WP6 | 0.011761 |
| WD4 | WP6 | 0.009397 |
| WD5 | WP6 | 0.008533 |
| WD5 | WP2 | 0.005519 |
| WD3 | WP6 | 0.004736 |
| WD4 | WA6 | 0.003978 |
| WD3 | WP2 | 0.003568 |
| WP2 | WP7 | 0.003555 |
| WD3 | WP7 | 0.003245 |
| WD4 | WP7 | 0.003139 |
| WP6 | WA6 | 0.003025 |
| WP4 | WV3 | 0.001880 |
| WD5 | WP7 | 0.001372 |
| WV12 | WV5 | 0.001288 |
| WV1 | WV8 | 0.001166 |

**Supplementary Figure S1**. Consensus plots from Structure analyses from K=2 to K=4 for all Palaearctic whimbrels.


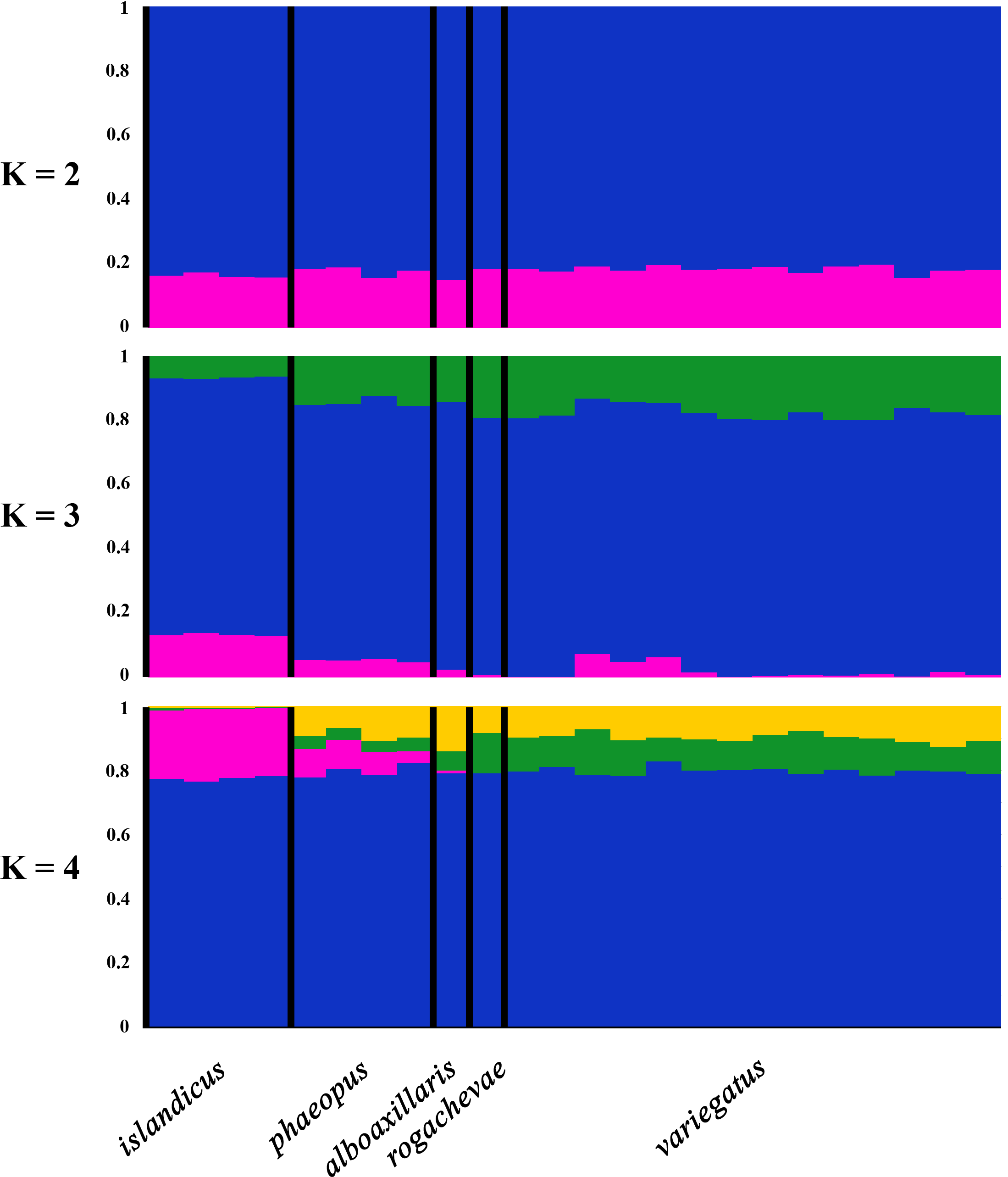


**Supplementary Figure S2**. Consensus plots from Structure analyses from K=2 to K=4 for Eurasian curlews. The ideal number of clusters is indicated by an asterisk.

**
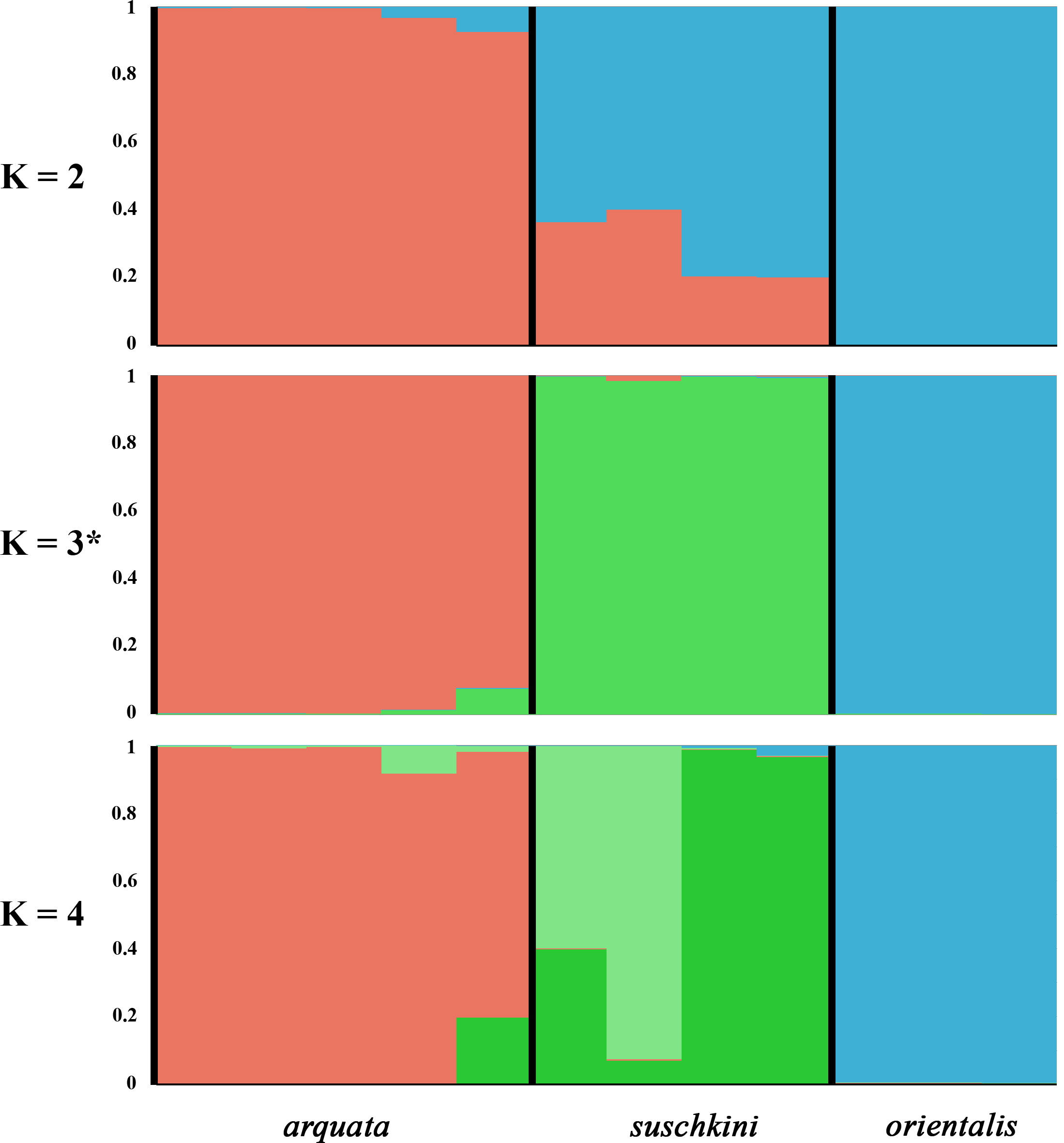
**

**Supplementary Figure S3**. Effective migration rates of Eurasian curlews from estimated effective migration surfaces analysis. The grey line outlines the polygon within which EEMS was performed. The two coloured areas are the only regions where EEMS detected non-significant deviations from effective migrations rates.**
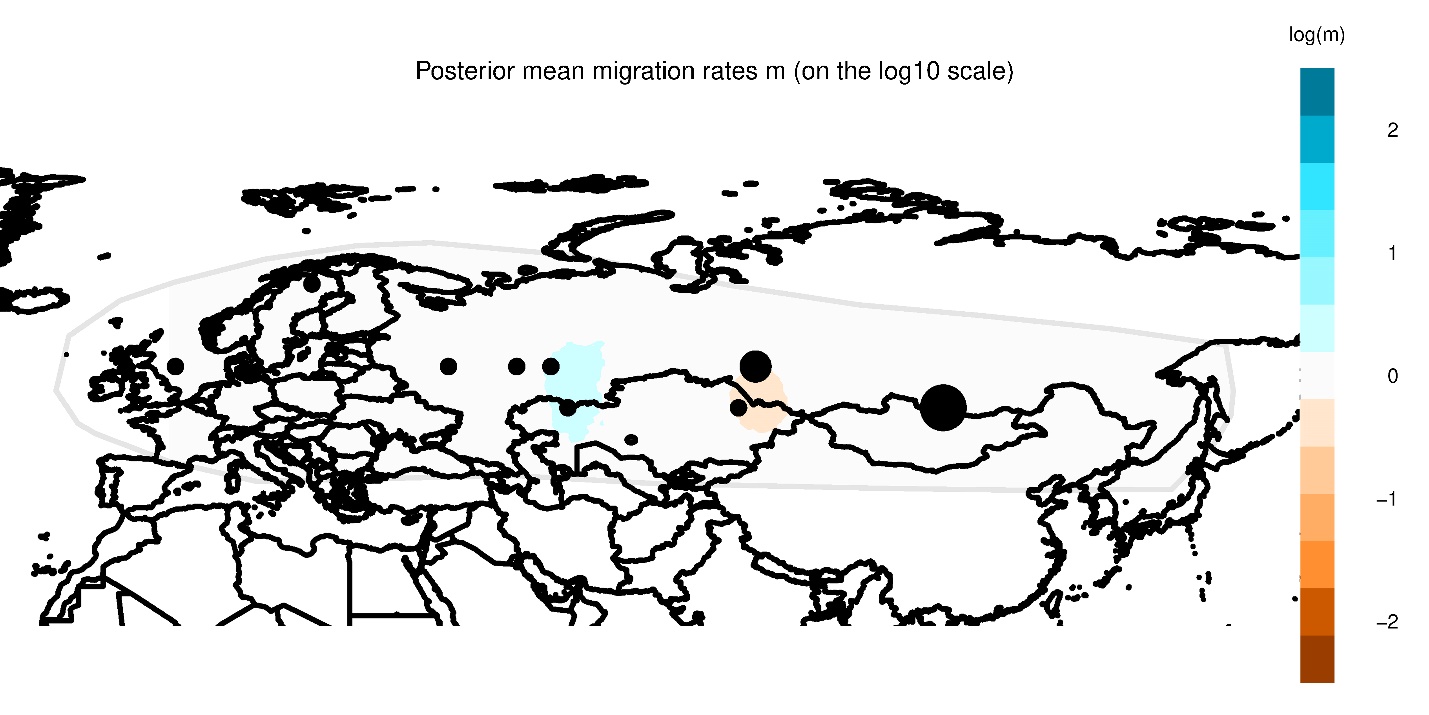
**
